# Supplementary material for: Are carbon emissions trading and green financial instruments synergistic? -Comprehensive quantitative research based on content analysis
Source: PLoS One. 2024 Mar 7;19(3):e0298601. doi: 10.1371/journal.pone.0298601 (PMC10919601; doi:10.1371/journal.pone.0298601)
Supplement: S1 Data — (ZIP) [file pone.0298601.s001.zip › Data/地级市Z_F工作报告.zip!/╕ú╜¿/╞╬╠∩╩╨/└┤╫╘╣1⁄2╓┌║┼í╛┬φ┐╦╩2╛▌═°í┐_2012.docx]

各位代表：

　　现在，我代表莆田市第五届人民政府向大会做工作报告，请予审议，并请市政协委员和其他列席同志提出意见。

加快追赶蓄势崛起的豪迈历程

2011年，在中共福建省委、省政府和中共莆田市委的正确领导下，市政府团结依靠全市人民，深入践行科学发展观，认真落实党中央、国务院《关于支持福建省加快建设海峡西岸经济区的若干意见》和《海峡西岸经济区发展规划》，大力实施“以港兴市、工业强市”发展战略，加快建设湄洲湾港口城市，圆满完成了市第五届人民代表大会第六次会议确定的目标任务。

国民经济较快增长，综合实力显著跃升。实现“超千争百促跨越”目标，预计全市生产总值增长15%，总量突破千亿元大关。财政总收入103亿元，增长32%。全社会固定资产投资750亿元，增长51%。社会消费品零售总额340亿元，增长17%。城镇居民人均可支配收入21800元，增长14%。农民人均纯收入9040元，增长18%。农林牧渔业总产值166亿元，增长0.4%。工业增加值突破500亿元，增长20%，工业对经济增长的贡献率超过60%。产业集群进一步发展壮大，竞争力明显增强。企业改制上市步伐加快。三棵树获得国家企业技术中心认定。新增26个发明专利、37个省著名商标和28个省名牌产品。获得“中国花蛤之乡”称号。

“五大战役”成效明显，重点建设有力推进。“五大战役”超额完成年度投资计划。“重点项目建设战役”完成投资432.7亿元，完成年计划116%；“新增长区域发展战役”完成投资250亿元，完成年计划133.6%；“城市建设战役”完成投资75亿元，完成年计划185.9%；“小城镇改革发展战役”完成投资181亿元，完成年计划142%；“民生工程战役”完成投资42.5亿元，完成年计划131.9%。列入全省重点跟踪管理的“三维”项目141个，总投资1953亿元，全年完成投资83亿元。福泉高速扩建工程莆田段、红星美凯龙家居城等项目投入运营，鞍钢冷轧、差别化纤维、妈祖城等重大工程建设及一批城市综合体项目进展顺利，乌溪中型水库、木兰溪后续防洪工程等重点前期项目取得突破。

城乡建设力度加大，宜居港城呈现新貌。向莆铁路、莆永高速、东吴港区泊位等一批交通运输项目建设加快推进。完成农村公路县道建设57公里，基础网络项目24公里，自然村项目160公里，安保工程310公里，改造危桥17座。新增公交线路5条、LNG公交车100辆、出租车200辆。实施城市绿化“八大工程”，城市建成区绿地率38.2%，绿化覆盖率43.4%，人均公园绿地面积11.8平方米，创建国家园林城市通过专家组现场考评。新增污水管网160公里，城市生活垃圾无害化处理率达98%以上。新农村建设有序推进，农村面貌持续改善。供水、供电、供气及邮电通讯等基础设施进一步完善。

改革开放纵深推进，体制机制更具活力。国企改革步伐加快，整合组建五大产业集团、三大投资公司。完成公路稽征系统成建制划转工作，农村公路管养体制进一步完善。强化发展服务中心职能，市级行政审批减少13.9%。创新农村工作机制，集体林权、土地经营权、海域使用权等综合改革稳步推进。深化文化、医药卫生体制改革，获得“全国文化体制改革工作先进地区”称号。成功承办第十三届中国·湄洲妈祖文化旅游节、第六届中国（莆田）海峡工艺品博览会等节庆活动。开展境内外各种招商推介等活动，合同利用外资8.6亿美元，实际利用外资8亿美元（历史可比口径），分别增长10%和4%。外贸进出口总额45亿美元，增长31.5%。对口支援扎实有效，四川灾后援建圆满完成。

和谐莆田加快建设，社会事业全面发展。科技事业稳步推进，连续四届获得“全国科技进步先进城市”称号。各类教育持续发展。莆田学院接受本科教学工作合格评估。湄洲岛被授予“海峡两岸交流基地”，并入选“世界节日活动之城”。电视剧《妈祖》开拍，《两岸一家亲》入选“唱响中国”十首优秀歌曲，莆仙戏鲤声剧团赴巴黎演出获得成功。城乡医疗卫生服务体系不断健全，乡镇卫生院、社区卫生服务中心设施全面提升。新农合政府补助标准提高到每人每年200元，参合率达99.8%。成功承办省第七届少数民族传统体育运动会和7项全国赛事。社会保障体系进一步健全，新型农村社会养老保险制度试点工作顺利开展，社会保险覆盖面不断扩大，城乡低保实现应保尽保。保障性住房建设力度加大，新建、改建各类保障性住房7551套。全年发放公积金贷款5.3亿元。人口计生工作完成省下达的责任指标，稳定低生育水平。节能减排任务有效落实，单位生产总值能耗降低3%，主要污染物减排任务全面完成。继续推进耕地保护和土地开发整理，实现耕地占补平衡。全面启动“六五”普法工作，着力建设“平安莆田”，社会和谐稳定。首次获得“全省文明城市”称号。创建“全国双拥模范城”工作顺利通过国家验收。民族宗教、外事侨务、保密、审计、统计、人事、旅游、档案、方志、气象、消防、边防和国防动员等事业取得新发展。

政府建设不断加强，执政能力有效提升。积极推进政府职能转变，完善政府决策机制，不断提高政府工作的科学化、民主化和法制化水平。认真抓好政风行风工作，完善政府绩效评估体系，开展“治庸懒提绩效促跨越”活动，各级政府工作效能不断提高。扎实推进惩防体系建设，严肃查处一批违纪违法案件。进一步提高应对突发公共危机事件、防范与化解社会矛盾的能力，有效应对“9.1”特大暴雨灾害。政府信用体系建设稳步推进。

各位代表，2011年经济社会发展成效显著，过去五年我们走过了极不平凡的历程。通过全市人民的生动实践和埋头苦干，兴化大地发生了广泛而深刻的变化，焕发出蓬勃生机和旺盛活力，湄洲湾港口城市展现出新的气象。

这是经济发展重大跨越的五年。预计全市生产总值年均增长超过15%，居全省第一。人均生产总值从15261元提高到37700元。财政总收入从37.3亿元增加到103亿元，年均增长22.5%。全社会固定资产投资累计突破2100亿元，年均增长35.3%。实际利用外资累计33亿美元（历史可比口径）。经济结构进一步优化，工业投资累计730 亿元，规模以上工业产值年均增长25.3%，总量突破1600亿元。十大产业集聚壮大，“五行”基地加快建设，工业强市成效显现。实现节能减排等约束性指标。中海油等20多家央属企业落户莆田。金鹰集团等一批跨国公司来莆投资兴业。福建LNG项目秀屿接收站、莆田燃气电厂等重大项目建成投产，石门澳临港产业园、抽水蓄能电站等重大项目加快建设，神华煤电等重大项目前期工作有力推进，为莆田发展注入强劲动力。

这是城乡面貌巨大变化的五年。全市公路总里程5566公里，新增376公里。高速公路通车里程70公里，新增24公里。高等级公路通车里程361公里，新增71公里。改造和建设农村公路2041公里。福厦铁路莆田段建成通车，结束莆田没有铁路的历史，铁路在建项目总里程86.3公里。城市框架向木兰溪畔延伸，极大拓展了城市发展空间，新增城市道路192公里。建成市体育中心场馆等一批标志性建筑。成功承办第十四届省运会。完成港口建设投资33亿元，港口生产性泊位增加到42个，吞吐能力超过4000万吨。建成大型金钟水库、木兰溪防洪一、二期工程，防灾减灾能力显著提高。解决不安全饮水170多万人，农村自来水普及率提高到89.6%。广播、电视覆盖率分别达99%和98%。城市污水处理率达80%以上。

这是人民群众得到实惠最多的五年。全市各级财政用于改善民生的投入逐年增长，完成54项市级惠民实事。累计新增城镇就业10.5万人，转移农村劳动力18万人，城镇登记失业率控制在3%以内。城镇基本医疗保险和新型农村合作医疗保险覆盖面分别达90%和99.8%。城镇居民人均可支配收入、农民人均纯收入年均增长12.2%和12.9%。全面实行城乡免费义务教育，基础教育普及程度显著提高，通过全省首批设区市“双高普九”验收。覆盖城乡的公共文化服务体系、公共卫生和医疗服务体系日益完善。妈祖信俗申遗成功。全面完成“五五”普法。精神文明和民主法制建设不断加强，社会和谐稳定局面得到巩固。

各位代表，五年来，我们在抢抓机遇中奋勇拼搏，在攻坚克难中奋力追赶，在先行先试中奋进跨越，谱写了莆田发展史上光辉灿烂的华彩乐章。我市成为海峡西岸经济区发展速度最快、发展质量最优、发展后劲最足和社会文明发展最好的地区之一，标志着莆田进入了蓄势崛起的崭新阶段。我们将在更高起点上，迈上新的跨越征程，创造更加美好的未来！五年的奋斗历程告诉我们：必须坚持科学发展观，抓住发展第一要务，加快发展，才能迈上发展新台阶。必须坚持从实际出发，走“以港兴市、工业强市”崛起之路，才能创造发展新局面。必须坚持解放思想，以新视野建新港城，以强投入促快发展，才能实现发展新跨越。必须坚持谋划长远，优化环境，埋头苦干，才能增创发展新优势。必须坚持以人为本，执政为民，着力改善民生，汇聚强大力量，才能形成发展新活力。

五年的巨大变化，是在省委、省政府和市委科学决策、坚强领导下不断开创的，是在全市人民和社会各界凝心聚力、团结拼搏下共同造就的，是在历届领导班子工作的基础上与时俱进、开拓创新持续形成的。在此，我代表市人民政府，向全市人民致以崇高的敬意！向人大代表、政协委员、各民主党派、工商联、各人民团体、无党派人士和社会各界，向驻莆部队、武警官兵和公安民警，向港澳台同胞、广大侨胞和海内外朋友，表示衷心的感谢！

五年的发展成就令人鼓舞，但前进道路上还面临不少困难和问题，政府工作还存在一些不足和薄弱环节。主要是：第三产业比重偏低。城市规划、建设和管理水平尚需提高。城乡之间、县区之间发展还不平衡。节能减排形势严峻。土地、资金、劳动力等要素保障能力还有待加强。违法建设现象尚未得到有效遏制。社会治安不尽如人意。安全生产压力较大。社会保障体系还不健全。政府职能转变还不到位，部分干部执行力、创新力、办事效率亟待提升。我们必须直面这些问题，认真有效加以解决，决不辜负全市人民的重托和期望。

乘势而上跨越发展的宏伟蓝图

今后五年，是莆田实现全面建设小康社会的关键时期。我们既面临难得的发展机遇，也面临严峻的现实挑战。海西战略的深入推进，莆田的区域优势、资源优势和市场优势进一步凸显，在海西发展格局中的地位更加突出。这一时期，是湄洲湾港口城市持续提升的关键阶段，我们要立足科学发展新实践，顺应人民群众新期待，谱写跨越发展新篇章。

新一届政府工作的指导思想是：高举中国特色社会主义伟大旗帜，以邓小平理论和“三个代表”重要思想为指导，深入贯彻落实科学发展观，大力实施海西《规划》，围绕“跨越发展、宜居港城”目标，深入实施“以港兴市、工业强市”发展战略，进一步解放思想，先行先试，加快转变经济发展方式，坚持“三化”并举，推进“三群”联动，拓展“三维”项目，注重发展实体经济，推进城乡一体化，切实保障改善民生，加快建设服务型政府，全面建设惠及全市人民的更高水平小康社会，努力建设富裕莆田、和谐莆田、文化莆田、生态莆田、幸福莆田。

今后五年经济社会发展的总体目标是：

经济实力跨步跃升。全市生产总值、财政总收入实现翻番，力争人均地区生产总值等主要经济指标达到全省平均水平，地区生产总值年均增长15%以上，财政总收入年均增长18%以上，全社会固定资产投资年均增长25%，社会消费品零售总额年均增长18%，外贸进出口总额年均增长10%。全面推进“跨越发展、宜居港城”建设，使莆田成为海西建设新的增长极。

宜居港城奋力崛起。按照“保护壶山兰水景观、突出荔林水乡特色、建设生态环保家园、打造滨海宜居城市”要求，积极创建国家级森林城市和国家级生态市，逐步形成“五山簇拥、四水相依、三湾环绕、二岛添辉、一心为源”的宜居城市风貌。加快推进城乡一体化步伐。提升中心城区，拓展东西两翼，面海环湾发展，保护生态资源，构建以主城区为核心、滨海新区和仙游县城为次中心的城市空间格局。全面开发湄洲湾、启动建设兴化湾、保护利用平海湾，港口吞吐量突破7000万吨，把莆田港建设成为海峡西岸具有深水中转和物资储备等多功能的综合性主枢纽港。

产业结构优化升级。加快构建以先进制造业和能源产业为主体、现代服务业和现代农业为支撑、三次产业协调发展的现代产业体系。在湄洲湾北岸重点建设铁矿石、木材、油品、天然气和煤炭“五行”基地，在兴化湾南岸重点培育装备制造、新能源、新材料和精细化工等新兴产业，在仙游枫亭重点培育石化产业。实现临港工业产值占规模工业产值的60%以上、重工业比重超过50%。做优做特第一产业，加快发展现代农业。做大海洋经济，着力发展海洋渔业、船舶修造和海洋运输等海洋产业。促进第三产业提速提质，力争第三产业增加值占GDP比重40%以上。力争高新技术产业增加值占GDP比重15%以上。

社会事业全面发展。全市人民受教育程度稳步提升，思想道德素质、科学文化素质和健康水平不断提高，社会主义民主法制更加健全。文化事业和文化产业加快发展。建成图书馆、博物馆、规划馆、科技馆、青少年宫和档案馆等一批公共服务设施。社会管理水平明显提高，社会更加和谐稳定。

幸福指数加快提升。城乡居民收入大幅度提高，城镇居民人均可支配收入年均增长11%，农民人均纯收入年均增长10%以上。力争城乡居民收入翻一番。就业持续增加。覆盖城乡居民的基本公共服务体系逐步完善，人民生活质量和水平不断提高，使莆田成为全市人民幸福生活的美好家园。

持续提升拼搏奋战的开局之年

2012年是新一届政府的开局之年，我们要全面贯彻落实中央、省经济工作会议和市第六次党代会精神，全力做好稳增长、控物价、调结构、惠民生、抓改革、促和谐各项工作，以优异的成绩迎接党的十八大胜利召开。按照“实现一二三一五、突破项目促跨越”的总体要求，经济社会发展的主要目标是：全市生产总值增长15%；规模以上工业产值确保增长20%，预期1940亿元，力争突破2000亿元；财政总收入增长24%，预期128亿元，力争达到133亿元，其中地方级财政收入增长19%，预期76亿元；全社会固定资产投资额增长23%，预期920亿元，力争突破1000亿元；社会消费品零售总额增长18%，预期400亿元；外贸进出口总额增长13%，预期51亿美元；实际利用外资增长6%，预期8.5亿美元；城镇居民人均可支配收入和农民人均纯收入分别增长12%左右；居民消费价格总水平涨幅控制在4%左右；城镇登记失业率控制在2.5%以内；人口自然增长率控制在7.8‰以内；完成单位生产总值能耗以及主要污染物排放年度控制目标。

为了实现以上目标，我们要重点抓好以下工作：

一、加快推进项目建设，拉动投资增长，创造发展新优势

着力攻坚“五大战役”。拓展战役的领域和内涵，把农林水利、“四绿”工程、城镇美化、防灾减灾、造福工程、饮水安全、医疗卫生和社会保障等领域的项目纳入战役实施范围。坚持迎难而上抓征迁，千方百计保资金，提速增效促审批，全力以赴，比学赶超，使“五大战役”成为推动“三化”并举、“三群”联动的有力抓手，成为扩大内需的有效途径，成为保障和改善民生的重要载体。

全面对接“三维”项目。坚持“项目为纲、投资为要”，凝心聚力抓项目，攻坚克难促投资，支撑经济跨越发展。加强与央企、外企和民企的洽谈对接，引进一批投资超10亿元、50亿元和100亿元的大项目。密切跟踪落实已签约的16个央企项目，继续推进神华煤电、北汽现代等8个在谈央企项目。深度对接林浆纸一体化等17个投资超亿美元的重大外资项目，全面落实已签约的港澳项目。进一步盯紧66个省级具备签约的民资项目。

推进交通枢纽建设。着力于大港口、大通道和大物流建设，推进以主枢纽港为龙头的交通运输体系建设。加快秀屿、东吴和兴化港区等一批深水码头建设，重点推进罗屿铁矿石码头、湄洲湾煤炭码头等20个大型泊位建设，续建湄洲湾三期航道，启动建设兴化湾南岸基础设施。建成莆永高速莆田段，加快建设沈海复线高速莆田段，开工建设湄洲湾至重庆快速通道莆田段。力争省道201线莆田段、确保省道202线涵江段竣工通车，加快建设石城等六条疏港路。加快推进向莆铁路、湄洲湾港口铁路支线建设进度。

二、加快落实惠农政策，做好“三农”工作，推进农业现代化

持续加大“三农”工作扶持力度。财政支出重点向农业农村倾斜，预算内固定资产投资重点用于农业农村基础设施建设，土地出让收益倾斜投向农业土地开发、农田水利和农村基础设施建设。创新农村工作机制，继续实施“三百”工程。实施新一轮造福工程。落实中央扶贫政策，支持偏远山区、贫困地区、老区和海岛等欠发达地区发展。

积极发展高效生态的现代农业。坚持用工业化理念发展农业，提高农业综合生产能力和市场竞争力。加快建设标准化、产业化、专业化生产基地，推进仙游县国家级现代农业示范区和东峤镇省级标准农田建设。大力发展精细农业、设施农业和高效生态农业，培育一批农业产业化龙头企业。实施农业名牌战略，引导企业申报无公害、绿色和有机农产品，健全农产品质量安全检验检测体系。抓好平海、上林和鳌屿3个渔港码头建设，发展海洋养殖、远洋捕捞。防控重大动植物疫病工作，确保畜牧业健康发展。

大力加强以现代水利为重点的农村基础设施建设。进一步完善农村水利、交通、通讯和电力等基础设施，加快建设“三纵三横”水资源供水网络，重点推进木兰溪防洪等十大水利工程，抓好中小流域治理，加快灌区续建配套与节水改造，加大水土保持生态修复力度，加强小型农田水利重点县、初级水利化县建设。实施新一轮农村饮水安全工程，有效保障39万人民群众饮水安全。

三、加快产业转型升级，优化产业结构，提升工业竞争力

着力发展临港产业和新兴产业。引进发展光电信息、生物医药、新材料和新能源等新兴产业。开工建设林浆纸一体化等项目，大力推进神华煤电一体化、核电小堆等项目前期工作，加快铁矿石、木材、油品、天然气和煤炭“五行”基地建设，推动临港产业迅速崛起。促进风力发电等60多家企业建成投产，新增产值60亿元以上。支持三棵树、佳通轮胎等企业增资扩产，新增产值120亿元。推动规模以下企业转规模以上企业，新增规模工业企业100家，新增产值50亿元。促进产能存量充分释放，增加产值120亿元。累计新增规模工业产值350亿元以上。

发展壮大特色优势产业。引导企业运用信息技术，推进科技创新，改造提升传统产业。加大制鞋技术研发力度，打造产品品牌，扩大市场份额。加大海峡纺织城等轻纺项目扶持力度，形成集纺织、印染、面料和服装于一体的产业链。鼓励英博雪津、闽中有机等重点食品企业扩大生产，大力发展饮料、果蔬、休闲食品和粮油深加工产品。贯彻落实中央、省对小微企业的支持政策，加大贷款担保、结构调整、节能减排和技术创新等方面的支持，提高市场竞争力。

大力推进自主创新。扎实开展科技创新型城市试点工作。大幅增加科技研发投入，借助“6·18”等平台，对接全国高校、科研院所，深入开展院地合作，加快海西研究院莆田分院建设步伐，推进企业自主创新。推动莆田高新区升格为国家级高新区。着力打造自主品牌，争创1-2个国家级区域品牌，2个国家级品牌产品、30个省级品牌产品。培育5家国家知识产权优势企业。推动发展由“要素驱动”向“创新驱动”转变，争创“莆田制造”和“莆田创造”双优势。

四、加快发展服务经济，扩大消费需求，培育经济增长点

重点发展现代服务业。培育发展金融业，争取一批商业银行落户莆田。积极发展会计、律师、审计、资产评估、规划设计、咨询和典当等服务业。加快发展总部经济、会展经济和楼宇经济，提升工业设计、科技信息服务、商务服务和服务外包等发展水平。加快发展现代物流业，推进物流园区、物流配送中心建设，引导传统运输、仓储企业向第三方物流企业转型。

巩固发展商贸业。培育引进大型商贸企业，扶持现有商贸企业做强做大，引导网贸企业健康发展。启动火车站站前商业片区开发，建成城区大型平战结合人防工程，开发繁荣商业中心。建设海峡国际商贸城等一批专业市场。加快建设香格里拉等一批星级酒店，提升宾馆酒店、餐饮服务业档次和品位。规范新增货车落籍和外挂车船回迁工作。

大力培育消费市场。推进限下企业转限上企业，新增限上企业200家，促进消费市场批发零售额的快速增长。继续推进“万村千乡市场工程”和农贸市场改造提升工程，推动农机、家电、汽车和摩托车下乡。进一步扩大内需，大力推动医疗保健、家政服务、健身娱乐和网络购物等新兴消费。建立信用风险分担机制，发展信贷消费，挖掘消费潜力，鼓励汽车、家电等耐用品消费。进一步规范房地产市场秩序。

持续提升旅游业发展水平。围绕建设国际旅游度假目的地，加强旅游品牌线路的策划、包装和宣传，着力打造特色旅游品牌。继续创建湄洲岛国家级风景名胜区和大九鲤湖景区，大力发展工艺美术文化、南少林禅武文化、荔林水乡都市休闲和闽中司令部红色主题等特色旅游，加快旅游基础设施和景区建设，发展旅游经济。

五、加快建设宜居城市，提升城市品质，展现城市新风貌

着力优化城乡规划。实施新一轮城市总体规划，主动对接福州、厦漳泉大都市发展规划，进一步优化港口、产业和城市空间布局，加快修编兴化湾南岸等重点区域发展规划，完善城区公共设施等专项规划。完成241个村庄规划。着力做好湄洲岛、木兰溪和火车站片区等重要地段规划和城市设计，力争用两到三年的时间，打造能够满足国际商务、旅游、休闲和体育等需求的中央商务区和街区。

提升城市建设水平。推进玉湖新城、木兰财富中心、木兰国际广场等城市综合体建设。加快霞林片区、妈祖城核心区和文甲码头等基础设施建设。修建木兰大道、壶公路等市政道路，新增市政道路76公里。打通城厢区南园路，延伸涵江人民街至荔涵大道。实施荔园路、天妃路等“白改黑”工程，新增沥青路面40公里。铺设学园路、南环城路地下电缆，新增地下电缆4公里。建成绶溪公园一期、泗华水上公园二期项目。修建九华山、天马山、壶公山、囊山、凤凰山和大蜚山6个生态森林公园。切实抓好供水供气、路灯夜景和公共消防设施建设。加快实施城市防洪排涝和水环境整治工程，大力抓好延寿溪、下磨溪和钟潭溪整治。持续推进棚户区、危旧房和城中村等旧城改造。加快各类开发园区整合、拓展和提升，促进功能完善的开发区向城市新区发展。

全面加强城市管理。加强公共场所周边环境卫生和经营秩序管理，加大对户外广告、门店招牌、交通标牌和房屋立面的规范整治力度，改善市容市貌。加大主次干道保洁力度，推进垃圾分类收集、清洁直运，提高无害化处理能力，着力打造清洁城市。强化建设工地施工管理，大力治理运输“滴撒漏”现象。加强道路交通、建筑施工和饮食服务业等噪声监管。综合治理交通拥堵，新增一批公交车辆，新建、改造一批停车场，大力整治违法占道、违法行驶，优化市民出行环境。实施数字惠民行动，推动智能交通、智能城管、智能医疗和智能教育等重点工程，方便居民衣食住行。

着力打造生态市。继续实施“四绿”工程，重点抓好铁路、高速公路和省道等沿线绿化，城市建成区绿地率达39%，绿化覆盖率达44%，人均公园绿地面积达11.9平方米。鼓励发展循环经济和低碳经济。全面落实“一岗双责”，严格节能减排奖惩措施，确保完成节能减排任务。大力推广清洁能源，积极实施“LNG替代计划”。加快建设市垃圾焚烧发电厂二期及3个污水厂工程，新增污水管网160公里，力争污水日处理能力达26万吨。持续开展农村家园清洁行动，农村生活垃圾无害化处理率达82%。强化饮用水保护措施，建立完善生态补偿机制。实施生物多样性保护工程，建成土海湿地公园，续建木兰溪源头、萩芦溪源头和老鹰尖自然保护区。

六、加快改革开放步伐，健全体制机制，开创发展新局面

推进城乡一体化综合改革试点建设。抓紧申报省城乡一体化综合改革试点市实施方案，积极争取出台相关配套政策。推动基础设施、公共服务向农村延伸，加快建设一批交通、给排水、天然气和输变电工程等基础设施。结合公路、铁路、水利和产业项目建设，高质量建设一批集中安置小区，逐步实现农村居住和生活条件与城市接轨。抓好4个省级、5个市级综合改革试点小城镇建设，提升小城镇功能。规范农村住房建设管理，建设一批中心村和新型社区。

大力提升开放水平。提高利用外资规模与质量，鼓励外商重点投资临海重化工、机械装备制造、船舶修造、环保、新能源、生物医药和电子信息等产业。支持外资企业上市，鼓励境外上市企业返程投资。加强在台湾、港澳和东南亚地区的专业化招商。转变外贸发展方式，积极组织企业参加中国-东盟博览会等境内外推介活动，大力支持鞋类、纺织、服装和食品等优势产品创建国际品牌，拓展市场，扩大出口。扶持壮大40家出口1000万美元以上的出口企业，引导有实力的外贸企业境外投资，参与境外资源性产品开发生产。鼓励进口天然气、木材、天然橡胶等资源性商品和先进技术设备。提升妈祖文化旅游节、艺博会水平，扩大影响力。

深化综合配套改革。加大违法占地违章建房整治力度，推进土地管理综合改革，创新土地节约集约利用机制、耕地保护模式，推广和谐征迁工作法。实施人才“双十”工程，引进和培养创新型科技人才和高技能人才。成立金融办，协调金融机构支持地方经济发展。健全企业信用担保，积极引进外资组建贷款担保公司。鼓励和引导企业改制上市。深化财政改革，科学制定预算编制，强化预算信息公开和执行管理，启动财政支出绩效评价试点工作，增加民生、三农、重点建设、产业转型升级和公共服务等投入。健全税收征管运行机制，强化第三产业税收稽查，保持税收稳定增长。继续深化国有资产管理、文化、医药卫生、教育、价格和农村综合改革。

加强对外合作交流。发挥妈祖文化的独特优势，深化莆台经贸、教育、文化、卫生和体育等交流交往。继续创建台商投资区。完善仙游国家级台湾农民创业园基础设施，继续引进台湾先进农业技术和名优品种。扩大莆台直航，力争莆台客货运直航常态化。加强与国际友好城市的交流合作。进一步提升与港澳经贸合作水平。发挥侨乡优势，积极引进侨资侨智。主动融入福州大都市区，促进福莆宁同城化。加强与向莆铁路沿线城市合作，拓展经济腹地。做好对口援建工作。

增强执行能力。强化执行意识，以执行出效益、创业绩、促发展。健全执行机制，明确执行任务，落实执行责任，加强执行监督，落实首问负责、限时办结和绩效管理等制度，提高办事效率。创新执行方法，加快电子政务建设，打造行政执行网络平台，实现政府、社会和群众之间高效良性互动，提高执行效率。改进执行作风，着力治庸治懒，实现快节奏运作、大力度推进、高效率落实。

提升服务效能。强化服务职责，立足发展大局，主动服务企业、服务基层、服务群众，让人民群众满意。转变服务职能，建立覆盖全民的公共服务体系，做好就业、社保、教育、医疗卫生和公用事业等公共服务，维护公平正义，让人民群众生活得更加幸福、更有尊严。创新服务方式，积极搭建各类综合服务平台，完善发展服务中心等窗口服务，深化行政审批制度改革，优化行政审批流程，精减审批事项，提高服务效能。

坚持廉洁从政。认真执行党风廉政建设责任制，扎实推进惩治和预防腐败体系建设，坚决惩处腐败分子，建设廉洁政府。深入开展纠风工作，坚决纠正损害群众利益的不正之风。完善电子监察系统，加强对政府投资项目和公共资金审计监察。厉行节约，勤俭办事，严格控制公务支出。

各位代表，新时期，新任务，新征程，引领我们锐意进取，激励我们奋勇拼搏。让我们在省委、省政府和市委的坚强领导下，不负重托，开拓创新，全面推进湄洲湾港口城市和世界妈祖文化中心建设，奋力谱写莆田科学发展、跨越发展的新篇章，为建设富裕莆田、和谐莆田、文化莆田、生态莆田、幸福莆田而努力奋斗!
